# Calcium-Sensing Receptor Antagonist NPS-2143 Inhibits Breast Cancer cell Proliferation, Migration and Invasion via Downregulation of p-ERK1/2, Bcl-2 and Integrin β1 and Induces Caspase 3/7 Activation

**DOI:** 10.34172/apb.2022.037

**Published:** 2021-02-01

**Authors:** Mohammad A.Y. Alqudah, Marwah Azaizeh, Aref Zayed, Leen Asaad

**Affiliations:** ^1^Department of Clinical Pharmacy, Jordan University of Science and Technology, Irbid, Jordan.; ^2^Department of Medicinal Chemistry and Pharmacognosy, Jordan University of Science and Technology, Irbid, Jordan.

**Keywords:** Breast cancer, Calcium-sensing receptor, NPS-2143, Caspase 3/7, Proliferation, Invasion

## Abstract

***Purpose:*** Calcium-sensing receptor (CaSR) has been associated with breast cancer metastasis tothe bone. Targeting chemoattractant factors, such as calcium, that are released in response tobone resorption could prevent metastasis and induce apoptosis of cancer cells. In the presentstudy, we investigated the potential caspase 3/7 activation following treatment with a CaSRantagonist, NPS-2143, in breast cancer cells. In addition, the effects of NPS-2143 on breastcancer cell proliferation, migration and invasion were assessed.

***
Methods:*** Colorimetric MTT assay was used to evaluate cell viability. Apo-one homogeneouscaspase-3/7 assay was used to measure caspase 3/7 activities in breast cancer cells. Cellmigration and invasion were assessed using scratch wound assay and matrigel invasionchambers, respectively. The protein expressions of p-ERK1/2, integrin β1 and Bcl-2 wereevaluated using western blotting.

***
Results:*** Our study revealed that NPS-2143 significantly reduced cell proliferation with halfmaximal (50%) inhibitory concentration (IC50) values of 4.08 and 5.71 μM in MDA-MB-231 and MCF-7 cells, respectively. NPS-2143 induced caspase 3/7 activation in MDA-MB-231 breastcancer cells which was accompanied with a remarkable reduction in the expression of Bcl-2antiapoptotic protein. NPS-2143 suppressed migratory and invasive abilities of MDA-MB-231cells with a significant reduction in the expression of p-ERK1/2 and integrin β1 proteins.

***
Conclusion:*** Our study confirms the ability NPS-2143 to suppress proliferative, migratory andinvasive effects of breast cancer cells which was accompanied by caspase 3/7 activation andsuggests the potential of NPS-2143 as a promising anti-cancer molecule in breast cancer.

## Introduction

Breast cancer, the most common type of cancer in women, commonly metastasizes to the bone.^1,2^ During bone resorption, several chemoattractant factors are released and they are considered the main driver of such selective metastatic destination to the bone.^1,3^ Therefore, characterizing molecular targets underlying this highly metastatic disease may lead to better therapeutics and improve such poor outcome. High levels of expression of calcium-sensing receptor (CaSR) have been reported in profiling studies of breast cancer gene expression in patients’ specimens as well as breast cancer cell lines specifically MCF-7 and MDA-MB-231 cells which has higher expression levels compared to nonmalignant breast cell lines.^4-7^ The CaSR is a class C/ G protein–coupled receptor that has a well-established physiologic role in regulating extracellular calcium levels and parathyroid hormone release.^8^

There is an increasing evidence from recent studies supporting the association of aberrant CaSR signaling in the growth and metastasis of several human cancers such as ovary, breast, kidney and prostate cancer.^8,9^ For instance, overexpression of a functional CaSR amplifies the osteolytic ability of metastatic breast cancer cells.^10^ In addition, small interfering RNA (siRNA) targeting CaSR could inhibit cell migration and proliferation in response to high Ca2+ concentration in breast cancer cell lines.^1,11^ Moreover, stimulation of CaSR could increase breast cancer cell proliferation by inducing the phosphorylation of extracellular signal-regulated kinases (ERK 1/2). Furthermore, stimulation of CaSR could protect breast cancer cells from caspase-independent apoptosis by hindering nuclear accumulation of apoptosis inducing factor (AIF).^11,12^ However, none of the previous studies have shown the consequent effects of CaSR inhibition on caspase activation which is a sign for caspase-dependent apoptosis. Therefore, the objective of this study was to evaluate the effect of *in vitro* CaSR inhibition via a calcilytic agent (NPS-2143) on breast cancer cell proliferation and consequent apoptosis via assessing the caspase 3/7 activation and the expression of the B-cell lymphoma 2 (Bcl-2) antiapoptotic protein. In addition, effects of NPS-2143 treatments on breast cancer cell migration and invasion were assessed.

## Material and Methods

### Cell lines, cell culture and drug treatment

Triple negative human MDA-MB-231 and hormone positive MCF-7 cells were purchased from the American Type Culture Collection (ATCC) (Manassas, VA, USA). Cell culture conditions were followed as described previously.^13^ Briefly, breast cancer cells were cultured in Roswell Park Memorial Institute (RPMI-1640) media supplemented with 10% fetal bovine serum (FBS) and 1% penicillin/streptomycin under a humidified atmosphere with 5% CO2 at 37°C. For drug treatment, the CaSR antagonist, 2-Chloro-6-[(2R)-3-[[1,1-dimethyl-2-(2-naphthalenyl) ethyl] amino-2-hydroxypropoxy] benzonitrile hydrochloride (NPS-2143 hydrochloride), purchased from Tocris Bioscience (Bristol, UK), was dissolved in dimethyl sulfoxide (DMSO) to produce a 10 mM stock concentration. DMSO final concentration was kept constant and never exceeded 0.1% in all treatment groups.

### MTT assay

Cell proliferation was evaluated using the colorimetric 3-(4,5-dimethylthiazol-2-yl)-2,5-diphenyl tetrazolium bromide (MTT) assay as described earlier.^14^ Breast cancer cells were seeded at 1×10^4^ cells/well (4 replicates/group) in 96-well plates. After attachment, cells were treated with several NPS-2143 concentrations (0.1, 0.5, 1, 2.5, 5, 10 µM) or DMSO for 48 hours. Cells were incubated with MTT solution for 4 hours at 0.5 mg/ml concentration. After dissolving the blue formazan crystals using DMSO, absorbance was measured at 490 nm and results were presented as a percentage of viable cells normalized to DMSO-treated cells according to the following equations:

% of viable cells in each well=(Abs _NPS-2143_ / Average of Abs _DMSO in 4 replicates_) * 100

*% of viable cells for each NPS-2143 concentration=Average of normalized % of viable cells in 4 NPS-2143 replicates*

### Caspase-3/7 assay

Activities of caspase-3/7 were assessed using the apo-one homogeneous caspase-3/7 assay (Promega, Madison, WI, USA) as previously described.^15,16^ After plating MDA-MB-231 cells in black 96-well plate, cell were incubated with certain concentrations of NPS-2143 for 48 hours. Following the cleavage of caspase substrate by caspase-3/7 activities, fluorescence was measured at 499/521 nm excitation/ emission. Medium with the Apo-ONE® Reagent was used as the blank value, and cells incubated without NPS-2143 but with Apo-ONE® Reagent were used as a negative control. Results were presented as a percentage of fluorescence in treated cells relative to control.

### Scratch wound and Invasion assays

Scratch wound assay was used to measure cell migration as described earlier.^14^ After seeding breast cancer cells until confluence in a 6-well plate, cells were synchronized for 6 hours. After that, a wound was done in the confluent monolayer of breast cancer cells. A serum free media containing the final NPS-2143 concentrations was added to the scratched cells and maintained until wounds became completely closed. Wounds’ closure was analyzed using NIH-Image J software. Antimigratory effect of drug treatment was normalized to the width of control cells.

For invasion assay, Corning BioCoat Matrigel Invasion Chambers (Corning Inc., Acton, MA, USA) were used as described previously.^13^ Cells were resuspended into serum-free media containing drug treatments and added to the top chambers. Bottom chambers were supplemented with 10% serum-containing media. About 48 hours later, the invading cells were fixed, stained and counted using NIH ImageJ software.

### Western Blot

Both breast cancer cells were seeded overnight at 5 × 10^4^ cells/well into 6-well plates. After attachment, cells were treated with several concentrations of NPS-2143 (0–10 μM). After 48 hours, media were discarded and cells were washed with ice-cold PBS. To lyse the cells, 150 μl radioimmunoprecipitation assay buffer containing protease inhibitor were added to each well for 30 mins on ice cube. Cell lysates were collected in Eppendorf tubes and centrifuged at 20,000 rpm for 30 mins. In all collected supernatants, proteins were quantified using the bicinchoninic acid (BCA) assay. Equal amounts of protein samples (35 μg) and 10 μL of protein ladder were loaded into polyacrylamide gel in tris-glycine buffer at 200 mv for 40 minutes at room temperature (RT). Gels containing separated proteins were transferred into nitrocellulose membranes on 200 mA for 1 hour. Afterward, membranes were blocked with 5% BSA in Tris-buffered saline with Tween 20 (TBST) for 2 hours at RT. Expression of relevant proteins was analyzed by overnight incubation with the following primary antibodies at 4°C: anti-p-ERK1/2 (Cell Signaling, #5726), anti-Bcl-2 (Cell Signaling, #3498), and anti-integrin β1 (Cell Signaling, #4706). Glyceraldehyde 3-phosphate dehydrogenase primary antibody (Cell Signaling, #5174) was used as a loading control in all experiments. After 3 times washing with TBST buffer, membranes were incubated with horseradish peroxidase-conjugated secondary antibodies for 2 hours at RT. After another 3 times washing with TBST, enhanced chemiluminescent (ECL) detection kit was used to visualize protein bands using the Montreal Biotech Fusion Pulse 6 imaging system (Montreal Biotec Inc., Dorval, Canada).

### Statistical analysis

GraphPad® Prism Statistical software (version 6, USA) was used to conduct statistical analysis as described previously.^14^ Statistical significance was detected using One-way analysis of variance (ANOVA) test and Tukey’s multiple comparison test. Half maximal (50%) inhibitory concentration (IC50) values were obtained by applying nonlinear regression curve fit analysis. *P* < 0.05 was considered statistically significant. For all experiments results are expressed as average ± SE of three independent experiments.

## Results

### CaSR inhibition suppresses the proliferation and induces caspase-3/7 activation in breast cancer cells

**Figure 1:**
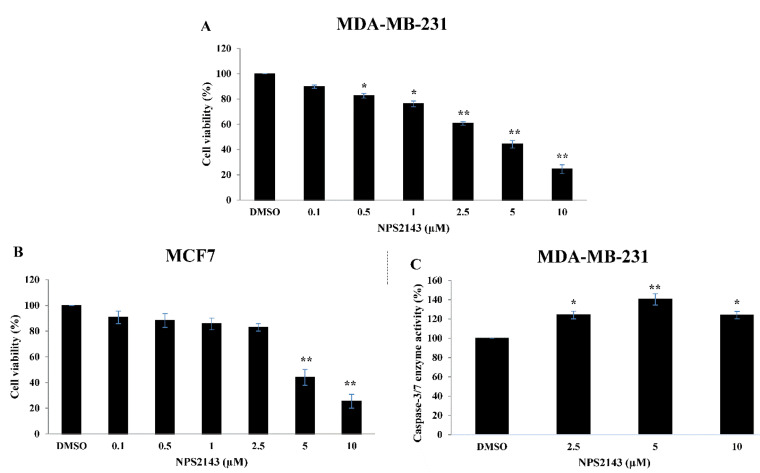
**
NPS-2143 treatment suppresses cell proliferation and induces caspase3/7 activity in breast cancer cells **. Cell viabilities of (A) MDA-MB-231 cells and (B) MCF-7 cells following NPS-2143 treatments were determined using MTT assay. (C) Effect of NPS-2143 treatment on caspase-3/7 activity in MDA-MB-231 cells. The data represent mean ± SE of three independent experiments. *indicates *P* < 0.05 and **indicates that *P* < 0.01 (ANOVA and Tukey’s multiple comparison tests).

The biological effects of various concentrations of NPS-2143 on breast cancer cell proliferation measured by *in vitro*MTT assay are indicated in Figure 1. Interestingly, the cell viability of both MDA-MB-231 and MCF-7 cells was reduced after 48 hours of NPS-2143 treatment in a dose-dependent manner. In more details, treatment with 0.5-10 µM of NPS-2143 significantly reduced MDA-MB-231 cell viability as compared to DMSO-treated controls (Figure 1a). In MCF-7 cells, 5-10 µM NPS-2143 significantly reduced MCF-7 cell viability as compared to DMSO-treated controls (Figure 1b). The IC_50_ values for NPS-2143 treatment were 4.08 ± 0.43 and 5.71 ± 0.73 μM in MDA-MB-231 and MCF-7 cells, respectively.

Since NPS-2143 treatment hindered growth of breast cancer cells, we further assessed the underlying molecular mechanism responsible for this growth suppression by measuring caspase-3/7 activities as a way to predict cellular apoptosis. The results indicated that the caspase 3/7 activity significantly increased following NPS-2143 treatment. In MDA-MB-231 cells, treatment with 2.5-10 µM of NPS-2143 significantly increased caspase 3/7 activity compared to DMSO-treated controls which could be a sign of caspase-dependent apoptosis (Figure 1c). However, we did not conduct caspase-3/7 assay on MCF-7 cells since these cells have variable expression of caspase-3.^17^

### CaSR inhibition suppressed the migration and invasion abilities of breast cancer cells

**Figure 2:**
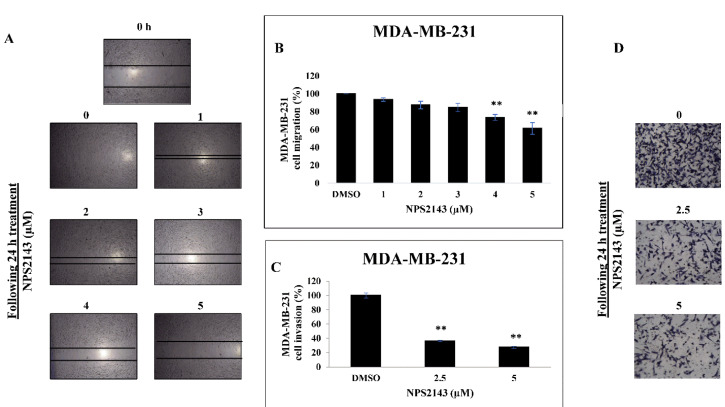
**
NPS-2143 treatment hinders cell migration and invasion of breast cancer cells.** (A) Scratch wound assay for MDA-MB-231 cells. Changes in cell migration per time relative to the baseline in MDA-MB-231cells treated with NPS-2143 or control was measured using ImageJ software. (B) Quantitative analysis of cell migration percentages following treatment of MDA-MB-231 cells with NPS-2143 (1-5 µM) compared to control treated cells. (C) Quantitative analysis of matrigel cell invasion assay. MDA-MB-231 cells treated with NPS-2143 or control were seeded into the upper chamber of the matrigel coated transwells. Using ImageJ software, invasive cells were quantified in four representative fields. (D) Representative images of invading MDA-MB-231 cells treated with NPS-2143 (2.5 or 5 µM) compared to control treated cells. **indicates that *P* < 0.01 (ANOVA and Tukey’s multiple comparison tests).

Based on our results, we hypothesized that CaSR could stimulate breast cell migration and invasion, a hallmark of invasive and metastatic breast cancer. For this experiment, we selected MDA-MB-231 cells which are known to be more migratory and invasive than MCF cells. Using an *in vitro*scratch wound assay, a complete closure of the inflicted wounds was observed in control wells while NPS-2143 treatment resulted in marked inhibition of MDA-MB-231 cell migration at 24 hours (Figure 2a). After performing quantitative analysis, results showed that treatment with 3-5 µM of NPS-2143 significantly reduced cell migration by (15-40) % in a dose dependent manner compared to DMSO-treated controls (Figure 2b). Most of the concentrations used in migration and invasion assays were below the IC_50_ value for MDA-MB-231 cells. In parallel, we confirmed the anti-migratory effects of NPS-2143 treatment by studying the ability of breast cancer cells to cross the basement membrane’s extracellular matrix (ECM) using matrigel invasion chambers as a way to mimic the *in vivo* process of cell invasion. The results indicate that NPS-2143 treatment had a notable inhibitory effect on MDA-MB-231 cell invasion (Figure 2c and d). Treatment with 2.5 and 5 µM of NPS-2143 significantly reduced cell invasion by more than 60% and 70%, respectively, as compared to vehicle-treated controls.

### CaSR inhibition effects on breast cancer cells were potentially mediated via ERK1/2, integrin β1 and Bcl-2 inhibition

**Figure 3:**
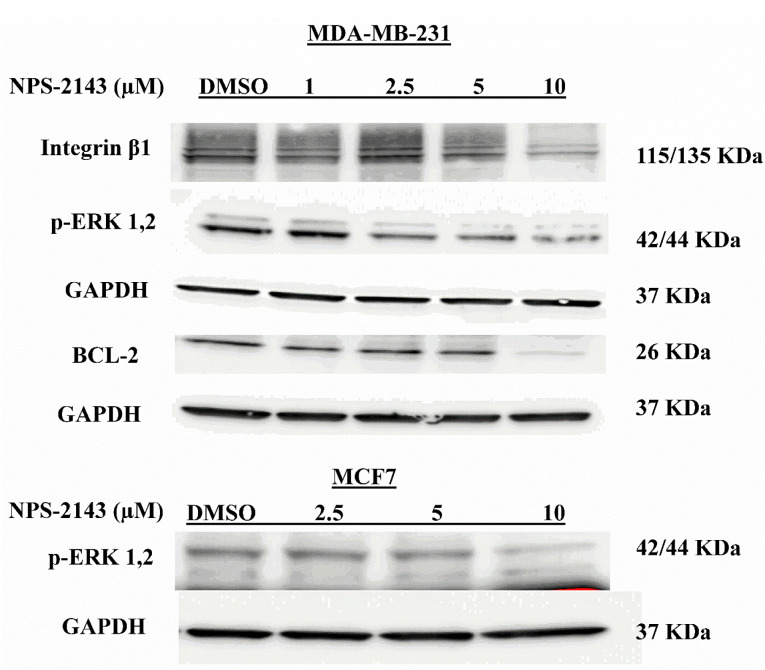
**
Influence of NPS-2143 treatment of breast cancer cells on p-ERK1/2, integrin β1 and Bcl-2 protein expressions **. Western blot analysis of the effect of NPS-2143 treatment for 48 hours. Downregulations of the relevant proteins was observed following NPS-2143 treatment.

To further elucidate the altered signaling pathways underlying the effects of NPS-2143 treatment on breast cancer cells, protein levels of key factors involved in cell proliferation, migration and apoptosis were assessed using immunoblot. Interestingly, our analysis revealed that NPS-2143 treatment significantly abolished ERK1/2 phosphorylation in both MDA-MB-231 and MCF-7 cells compared to DMSO-treated controls (Figure 3). In addition, NPS-2143 treatments resulted in downregulation of the antiapoptotic and caspase activator protein, Bcl-2 and the key factor in cell adhesion, integrin-β1 in MDA-MB-231 cells compared to DMSO-treated controls (Figure 3).

## Discussion

Oncogene overexpression is a well-known ubiquitous mechanism of tumorigenesis.^18^ Several studies have shown that CaSR is overexpressed in breast cancer in patients’ specimens and in malignant cell lines compared to nonmalignant breast cells.^6,7^ More recent studies have implicated CaSR in breast cancer metastasis to the bone due to calcium stimulation of these receptors.^10^ In this study, we found that NPS-2143 treatment had significant anticancer effects on breast cancer cells which was represented by suppression of cell proliferation and stimulation of caspase-dependent apoptosis.

In the present study, we explored the potential anticancer effects of the small molecule (NPS-2143) in breast cancer cells. Our results elucidate the pivotal role of NPS-2143 treatment in suppression of breast cancer cell proliferation, migration and invasion. In addition, activation of caspase-dependent cellular apoptosis was also observed. These effects were accompanied by downregulation of major aberrant signaling pathways in breast cancer such as p-ERK1/2, integrin β1 and Bcl-2, which are all often altered in breast cancer. Our data are in parallel with previous studies about the oncogenic role of CaSR in breast cancer, melanoma, prostate, kidney, colon and gastric cancers.^12,19-25^ NPS-2143 treatment resulted in significant suppression of breast cancer cell viability and the effect of growth suppression was significantly started at low NPS-2143 concentration (0.5µM) and maintained to the maximum effect at 10 µM. Our data showed that NPS-2143 treatment could abolish malignant breast cancer cell survival demonstrating its antiproliferative effect on breast cancer.

Kim et al have shown that activation of the CaSR through high extracellular calcium results in breast cancer cells resistance to caspase-independent apoptosis by hindering nuclear accumulation of AIF.^11^ In our condition, we reported that NPS-2143 treatment of breast cancer cells resulted in caspase-dependent apoptosis as shown by activation of caspase3/7. However, the increase in caspase3/7 activities started to decline at high concentrations (10 µM) which could be due to the dominance of caspase independent apoptosis at high concentrations. We were concerned to identify possible mechanisms for stimulation of such apoptosis following NPS-2143 treatment and found that NPS-2143 treatment resulted in downregulation of the anti-apoptotic Bcl-2 protein that is known to activate caspase pathway under cellular stress. In fact, previous studies have shown a direct regulation of Bcl-2 and the downstream caspases by CaSR signaling.^23^ For instance, NPS-2143 treatment has been reported to cause a promoting effect on apoptosis via induction of caspase 3 activities and inhibition of Bcl-2 protein expression in gastric cancer cells.^22,23^ Furthermore, NPS-2143 treatment of melanoma cancer cells has been shown to inhibit proliferation and induce apoptosis via upregulation of caspase 3 and downregulation of Bcl-2 protein expressions.^22^ Together, these results strongly indicate the caspase-dependent pro-apoptotic effect of NPS-2143 in breast cancer that occur at least via modulation of Bcl-2 and downstream caspase dependent components.

The role of CaSR in cancer cell migration and invasion has been studied before.^1,21,24^ Previous studies have shown that CaSR activation and signaling by elevated calcium concentrations resembling those found near resorbing bone could promote MDA-MB-231 cellular migration which is a breast cancer cell line with high metastasizing potential into bone compared to cells with a lower bone-metastasizing behavior such as MCF7 cells.^1^ These effects have been shown to be mediated via ERK1/2, phospholipase C beta 1 (PLC-β1) and mitogen-activated protein kinase. Interestingly, Hernández-Bedolla et al have shown that CaSR expressed in metastatic MDA-MB-231 cells could promote constitutive interleukin 6 (IL-6) secretion at basal level and thus mediate the process of metastasis. On the other hand, NPS-2143 treatment at high calcium concentrations has been found to inhibit IL-6 secretion and thus prevent cellular metastasis.^26^ Similarly, our study revealed anti-migratory and anti-invasive effects of NPS-2143 treatment in MDA-MB-231 cells. In concordance with previous studies, these effects were mediated via inhibition of ERK1/2 phosphorylation and integrin β1 downregulation. In renal cell carcinoma, CaSR promotes cell migration via enhancing ERK1/2 and other factors’ activities.^24^ In thyroid cancer cells, the CaSR induces cell migration via interaction with β1 integrin.^21^ Collectively, these findings confirm the anti-migratory as well as the anti-invasive effects of NPS-2143 in breast cancer.

## Conclusion

Our findings revealed the ability of NPS-2143 treatment to alter the aggressive behavior of breast cancer cells in terms of proliferation, invasion and resistance to apoptosis. This modulatory effect of NPS-2143 treatment can be at least attributed to its effects on the major altered signaling pathways in breast cancer such as p-ERK1/2, integrin β1 and Bcl-2, which are all often altered in breast cancer. In addition, NPS-2143 treatment could abolish the survival of breast cancer cells in terms of inducing caspase-dependent apoptosis.

## Acknowledgments

We are grateful to Jordan University of Science and Technology for funding this project (Deanship of Research, Grant# 92/2017).

## Ethical Issues

None.

## Conflict of Interest

None declared.
